# Effect of the Combination of Gelam Honey and Ginger on Oxidative Stress and Metabolic Profile in Streptozotocin-Induced Diabetic Sprague-Dawley Rats

**DOI:** 10.1155/2014/160695

**Published:** 2014-04-16

**Authors:** Nur Fathiah Abdul Sani, Levin Kesu Belani, Chong Pui Sin, Siti Nor Amilah Abdul Rahman, Srijit Das, Thent Zar Chi, Suzana Makpol, Yasmin Anum Mohd Yusof

**Affiliations:** ^1^Department of Biochemistry, Faculty of Medicine, Universiti Kebangsaan Malaysia, Jalan Raja Muda Abdul Aziz, 53000 Kuala Lumpur, Malaysia; ^2^Faculty of Medicine, Universiti Kebangsaan Malaysia Medical Center (UKMMC), Jalan Yaacob Latif, Cheras, 56000 Kuala Lumpur, Malaysia; ^3^Department of Anatomy, Faculty of Medicine, Universiti Kebangsaan Malaysia, Jalan Raja Muda Abdul Aziz, 53000 Kuala Lumpur, Malaysia

## Abstract

Diabetic complications occur as a result of increased reactive oxygen species (ROS) due to long term hyperglycaemia. Honey and ginger have been shown to exhibit antioxidant activity which can scavenge ROS. The main aim of this study was to evaluate the antioxidant and antidiabetic effects of gelam honey, ginger, and their combination. Sprague-Dawley rats were divided into 2 major groups which consisted of diabetic and nondiabetic rats. Diabetes was induced with streptozotocin intramuscularly (55 mg/kg body weight). Each group was further divided into 4 smaller groups according to the supplements administered: distilled water, honey (2 g/kg body weight), ginger (60 mg/kg body weight), and honey + ginger. Body weight and glucose levels were recorded weekly, while blood from the orbital sinus was obtained after 3 weeks of supplementation for the estimation of metabolic profile: glucose, triglyceride (TG), superoxide dismutase (SOD), catalase (CAT), glutathione peroxidase (GPx), reduced glutathione (GSH): oxidized glutathione (GSSG), and malondialdehyde (MDA). The combination of gelam honey and ginger did not show hypoglycaemic potential; however, the combination treatment reduced significantly (*P* < 0.05) SOD and CAT activities as well as MDA level, while GSH level and GSH/GSSG ratio were significantly elevated (*P* < 0.05) in STZ-induced diabetic rats compared to diabetic control rats.

## 1. Introduction

Diabetes mellitus (DM) is characterized by chronic hyperglycaemia caused by defects in insulin secretion, insulin action, or both, resulting in impaired function in carbohydrate, lipid, and protein metabolism. The total number of people with diabetes globally is expected to rise from 171 million in 2000 to 366 million by the year 2030 [1]. In Malaysia, the First National Health and Morbidity Survey (NHMS I) conducted in 1986 reported a prevalence of diabetes of 6.3% and in the Second National Health and Morbidity Survey (NHMS II) in 1996; this had risen to 8.3% [2]. According to the third survey (NHMS III) the overall prevalence of diabetes is 11.6% in individuals above 18 years and 14.9% in those above 30 years [3]. Alarmingly, the fourth survey conducted in 2011 (NHMS IV) showed that the prevalence of diabetes in individuals of 18 years and above had risen to 15.2% [4].

According to American Diabetes Association, diabetes mellitus (DM) is a group of metabolic diseases characterized by hyperglycemia resulting from defects in insulin secretion, insulin action, or both. Type I DM or juvenile-onset diabetes is caused by an autoimmune destruction of beta cells of the pancreas causing no production of insulin. Type II DM or adult onset diabetes involves a combination of reduced insulin production with increased cell insulin resistance. The third type of DM is known as gestational DM, which occurs during pregnancy [5]. Although the exact mechanism of how DM may cause oxidative stress is not known, it is highly probable that long term hyperglycaemia causes autooxidation of glucose which will eventually lead to microvascular and macrovascular complications [6]. Oxidative stress results from the inability of endogenous antioxidants to neutralize the abnormally high level of reactive oxygen species (ROS) which can react with and damage complex cellular molecules such as lipids, proteins, and DNA [6]. This results in changes in the activity of endogenous antioxidant enzymes, such as superoxide dismutase (SOD), glutathione peroxidase (GPx), and malondialdehyde level (MDA), as well as DNA damage in DM patients [7, 8]. Antioxidant enzymes and dietary compounds such as vitamins C, E, and flavonoids may have an effect in reducing the increased serum glucose level in DM and secondary complications of neuropathy and nephropathy as well as macrovascular complications of stroke and cardiovascular problems in people with longstanding uncontrolled DM [9].

In the past decade, rigorous research had been performed to find out the best way to overcome the complications as observed in DM, either by modern or alternative medicine. In Malaysia, research on herbal medicine in alleviating DM using animal and human studies has gained wide momentum in the past few years [10]. A formulated polyherbal drug consisting of extract of root of* M. paradisiaca*, seed of* T. indica*, seed of* E. jambolana,* and leaf of* C. indica *was found to have a potent antidiabetogenic efficacy in STZ-induced diabetic rats by reducing oxidative stress and carbohydrate metabolic profile and enzymes activities such glucose-6-phosphatase, lactate dehydrogenase, and hexokinase as well as glucose-6-phosphate dehydrogenase in liver, skeletal muscle, and cardiac muscle towards the normal range [11]. Aizzat et al. [12] reported a reduction in lipid peroxidation as measured by MDA levels and reduction in DNA damage evaluated by Comet assay in STZ-induced diabetic rats when supplemented with 150 mg/kg body weight* Chlorella vulgaris*.

For centuries, honey has been used as a substitute for sugar as well as providing medicinal benefit. In the past decade, numerous biological and nutritional effects of honey have been reported such as antimicrobial, antioxidant, antiviral, antiparasitic, anti-inflammatory, antimutagenic, anticancer, and immunosuppressive activities [13, 14]. In addition, Yao et al. [15] showed that higher concentration of gelam honey (5 g/kg body weight) reduced oxidative damage better than lower concentration (2.5 g/kg body weight) in young and middle aged rats by modulating antioxidant enzyme activities. Ginger, which is an underground rhizome of plant* Zingiber officinale* belonging to the family Zingiberaceae, was reported to have hypoglycaemic, anti-inflammatory, antitumorigenic, and antioxidant effects in* in vitro* and* in vivo* studies [16–20]. However, to the best of our knowledge, no previous studies have explored the antidiabetic and antioxidant properties of the combination of gelam honey and ginger on the oxidative stress and metabolic profile of STZ-induced diabetic rats, although a study by Patel et al. [21] reported the synergistic effect of the combination of honey and ginger on the antibacterial property on isolates of extracted carious teeth during orthodontic treatment. Thus, we embarked on this project to evaluate whether the combination effect of gelam honey and ginger would synergize in their actions in reducing oxidative stress and alleviating the complications of diabetes.

## 2. Materials and Methods

### 2.1. Animals and Diet

A total of 60 male Sprague-Dawley rats were obtained from by Animal Care Unit, Universiti Kebangsaan Malaysia (Bangi, Malaysia). The study was approved by the Animal Ethics Committee of the Faculty of Medicine, Universiti Kebangsaan Malaysia (FP/BIOKIMIA/2012/YASMIN/26-SEPTEMBER/463-SEPTEMBER-2012-AUGUST-2013). Prior to the experiment, rats weighing 200–250 gm were randomly chosen. They were housed individually in polycarbonate cages at a temperature of 22°C, with a 12-hour light-12-hour dark-cycle and was quarantined for one week and fed with a standard lab chow and water* ad libitum*. The rats were divided into two groups which were control (28 rats) and diabetic groups (32 rats). The control group was further divided into four groups: (i) control group fed with normal lab chow, (ii) control group fed with gelam honey (2.0 g/kg body weight), (iii) control group fed with ginger (60 mg/kg body weight), and (iv) control group fed with gelam honey and ginger (2.0 g/kg body weight honey + 60 mg/kg body weight ginger). Diabetes was induced by a single intramuscular injection of streptozotocin (STZ) (55 mg/kg body weight). STZ was prepared by dissolving it in cold 0.9% NaCl. It was injected into overnight fasting rats through the thigh muscle bulk. The rats were observed closely for symptoms of DM such as polydipsia, polyuria, and weight loss. Blood samples were obtained from rats' tail vein 48 hours after STZ injection. If blood glucose level was >14 mmol/L, rats were considered diabetic. The diabetic rats were then divided into four groups, that is, (i) STZ-induced diabetic rats fed with normal chow food, (ii) STZ-induced diabetic rats fed with gelam honey (2.0 g/kg body weight), (iii) STZ-induced diabetic rats fed with ginger (60 mg/kg body weight), and (iv) STZ-induced diabetic rats fed with gelam honey and ginger. The blood glucose level was taken once weekly starting from week 0 to week 3. After 3 weeks, blood samples were obtained from periorbital sinus vein for the evaluation of metabolic and oxidative markers such as blood glucose, triglyceride (TG), superoxide dismutase (SOD), glutathione peroxidase (GPx), catalase (CAT) activities, glutathione peroxidase (GPx), reduced glutathione (GSH): oxidized glutathione (GSSG), and malondialdehyde (MDA).

### 2.2. Ginger Extract by Hot Compressed Water Extraction (HCW)

An amount of 75 gm of ground ginger was weighed before being loaded into a covered stainless steel mesh cylinder and placed into the extraction cell of fabricated hot compressed water extraction** (**HCW) equipment. Approximately 700 mL of distilled water was added into the extraction cell and was covered securely with stainless steel lid. N_2_ gas was then used to purge out air and dissolved oxygen gas from the cell by letting the gas passing through the cell and out using release valve. The temperature was set up according to the design of experiment from 100 to 200°C by increasing 10°C at each experiment in 30 min of extraction time. All experiments were carried out at constant pressure of 3.5 MPa. Once the extraction process was completed, the extractant was immediately transferred into the cooling cell. Ginger extract was collected and stored at 4°C.

### 2.3. Honey

Malaysian monofloral gelam honey is produced by* Apis mellifera* bees and the major nectar and pollen collected by the bees is from the plant* Melaleuca cajuputi Powell*, which is known locally as the “gelam tree.” It was provided by the National Apiary, Department of Agriculture, Batu Pahat, Johor, Malaysia.

### 2.4. Preparation of Blood Sample for Enzyme Assays

Blood obtained from the periorbital sinus vein was collected in test tubes with heparin to prevent blood coagulation, and the plasma was separated. The blood samples were then rinsed with the same volume of 0.9% normal saline (NaCl) and centrifuged at 3000 rpm for 10 min at 4°C. The upper layer was removed and the above procedure was repeated with 0.9% NaCl until it became clear. The lower layer, termed hemolysate, was then used for the antioxidant enzyme assays. The upper layer, termed supernatant, was used for lipid profile test.

### 2.5. Determination of Body Weight

The rats were weighed weekly from the start of the study (i.e., at the end of one week of acclimatization to the laboratory environment), with a laboratory scale (Harvard Trip Balance, Florham Park, NJ, USA) to the nearest gram.

### 2.6. Determination of Glucose Level

Fasting blood glucose level was estimated by glucometer (Accuchek Instant, Germany) using a drop of blood taken from the tail vein of rats. Glucose estimation was made weekly throughout the period of study.

### 2.7. Triglyceride (TG) Level Measurement

Blood triglyceride level of animals was estimated by Reflotron (Boehringer-Mannheim, Germany) using plasma from the processed blood. The fresh whole blood taken from the periorbital sinus of rats was centrifuged at 3000 rpm, 4°C for 10 min. The plasma was then obtained from the processed blood. An amount of 30 *μ*L of plasma was placed on the Roche Reflotron Triglyceride strip and then placed into the Reflotron machine to be analysed. The amount of triglyceride is expressed as mmol/L.

### 2.8. Superoxide Dismutase (SOD) Activity Measurement

Superoxide dismutase activity was measured according to the method of Beyer Jr. and Fridovich [22]. Briefly, 1.0 mL aliquot of a mixture containing 0.1 mM phosphate buffer pH 7.8, 57 *μ*M nitro blue tetrazolium (Sigma, St Louis, USA), 9.9 mM L-methionine (Sigma, St Louis, USA), and 0.025% Triton-X (Sigma, St Louis, USA) was pipetted into test tubes. Then, 20 *μ*L of hemolysate and 10 *μ*L of a solution containing 4.4 mg/100 mL riboflavin (Sigma, St Louis, USA) were added into the mixture. The tubes were illuminated for 7 min in an aluminium foil-lined box containing two 20-W Sylvania GroLux fluorescent lamps. Absorbance was then measured at a wavelength of 560 nm. One unit of SOD was defined as the amount of enzyme required to inhibit nitro blue tetrazolium reduction by 50% per min per mL hemolysate. Enzyme activity was expressed as units per mg of Hb (U/mg Hb).

### 2.9. Glutathione Peroxidase (GPx) Activity Measurement

GPx was determined by the method developed by Paglia and Valentine [23]. The reaction mixture contained 0.05 M phosphate buffer pH 7.0, 8.4 mM NADPH (Sigma, St Louis, USA), 1.125 M sodium azide (Hopkin & William, England), 5 mM reduced glutathione (GSH), NADPH (Sigma, St Louis, USA), and 3 U/mL glutathione reductase (Sigma, St Louis, USA). The hemolysate was prepared by adding an equal volume of distilled water to the RBC pellet and was allowed to stand for 1 h at 4°C. Then four parts by volume of distilled water were added. Finally, double strength Drabkin's reagent (Eagle Diagnostics, Japan) was added to yield the final hemolysate. The reaction was initiated by adding 0.1 mL of 2.2 mM H_2_O_2_ (Merck, Darmstadt, German). The conversion of NADPH to NADP+ was followed by measuring the change in O.D/min at 340 nm. One unit of GPx was defined as the amount of enzyme required to oxidize 1 *μ*mol NADPH/min per mL hemolysate. Enzyme activity was expressed as milliunits per mg of Hb (mU/mg Hb).

### 2.10. Catalase (CAT) Activity Measurement

CAT was assayed by the method of Aebi [24]. The reaction mixture consisted of 50 mM phosphate buffer pH 7.0 and 30 mM hydrogen peroxide (H_2_O_2_). An amount of 0.025 mL of haemolysate was added with 12.5 mL of phosphate buffer to make a solution with 500x dilution. The reaction was started with the addition of 1.0 mL 30 mM H_2_O_2_ to 2.0 mL of hemolysate and the absorbance at 240 nm was read at room temperature against a blank containing 2.0 mL haemolysate plus 1.0 mL phosphate buffer. One unit of catalase enzyme was defined as the amount of enzyme which liberates half the peroxide oxygen from H_2_O_2_ solution in 30 s at room temperature. Enzyme activity was expressed as units per mg of Hb (U/mg Hb). Hemoglobin in the hemolysate was measured by using Hemoglobin kit (Eagle Diagnostics, Japan).

### 2.11. Total Glutathione (TGSH), Reduced Glutathione (GSH) Level, and Oxidized Glutathione (GSSG) Level Measurement

TGSH, GSH, and GSSG were determined by the method of Tietze [25] and Griffith [26] with some modifications. For the measurement of total TGSH content, 30 *μ*L of plasma was incubated with 30 *μ*L of 5,5′-dithiobis(2-nitrobenzoic acid) (DTNB) (Sigma, St Louis, USA), phosphate buffer containing EDTA, glutathione reductase (250 units/mL), and NADPH. Optical densities of the chromophores formed were monitored at 405 nm for 4 minutes. For GSSG assay, 100 *μ*L of plasma is derivatized by adding 2 *μ*L of 2-vinylpyridine (Sigma, St Louis, USA) and incubated for 30 min at 25°C. The assay was performed in a similar manner as total GSH equivalent. Protein concentration was determined using the Bradford Protein Assay Kit (Bio-Rad, USA). Reduced glutathione (GSH) was calculated by using the formula

(1) $$\begin{array}{c} \text{TGSH}\;\text{equivalent}=2\;\text{GSSG}+\text{GSH}. \end{array}$$

### 2.12. Malondialdehyde (MDA) Level Measurement

Plasma malondialdehyde (MDA) was determined using high performance liquid chromatography (HPLC) with photo diode array detector (Shimadzu, Japan) as described by Pilz et al. [27] with some modifications. Briefly, samples (50 *μ*L) were mixed with 200 *μ*L of 1.3 M NaOH and incubated at 60°C for 60 min. After cooling the mixture, 100 *μ*L of 35% HCIO_4_ was added and centrifuged at 10,000 g for 10 min at 4°C. The supernatant of the samples (300 *μ*L) was transferred into 1.5 mL of HPLC tube. 50 *μ*L of 5 mM DNPH solution was added into the mixture and incubated for 30 min at room temperature. Then, 40 *μ*L samples were injected into the HPLC. The amount of MDA is expressed as concentration of MDA in nmol per mL plasma.

### 2.13. Statistical Analysis

Data was analysed using SPSS package (version 21). Results refer to mean ± SD. Statistical evaluation was assessed using analysis of variance (ANOVA). A *P* < 0.05 was considered significant.

## 3. Results 

### 3.1. Effect of Gelam Honey, Ginger, and Their Combination on Body Weight in STZ-Induced Diabetic Rats

As shown in Table 1, diabetic control rats showed reduction in body weight as compared to normal control rats, which further decreased during the experimental period. Treatment with gelam honey, ginger, and their combination did not improve the body weight after 3 weeks.

### 3.2. Effect of Gelam Honey, Ginger, and Their Combination on Blood Glucose Level in STZ-Induced Diabetic Rats

**Figure 1:**
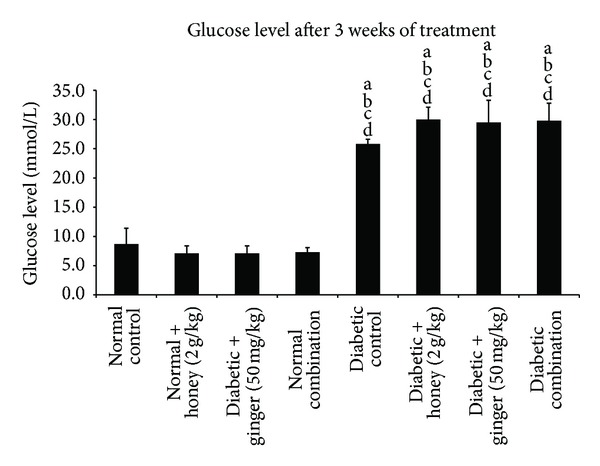
Blood glucose level after 3 weeks of treatment in normal and STZ-induced diabetic rats with various treatments. Data are expressed as mean ± SD. a: significant (*P* < 0.05) compared to normal control; b: significant (*P* < 0.05) compared to normal + gelam honey; c: significant (*P* < 0.05) compared to normal + ginger; d: significant (*P* < 0.05) compared to normal combination.

All rats injected with STZ developed severe diabetes as indicated by serum glucose concentrations >14 mmol/L over the period of 3 weeks. The diabetic control rats had significantly elevated blood glucose level compared to normal control rats at the end of week 3. Treatment of the diabetic rats with honey, ginger, or their combination did not significantly reduce the glucose levels when compared with the diabetic control group (Figure 1).

### 3.3. Effect of Gelam Honey, Ginger, and Their Combination on Triglyceride (TG) Level in STZ-Induced Diabetic Rats

**Figure 2:**
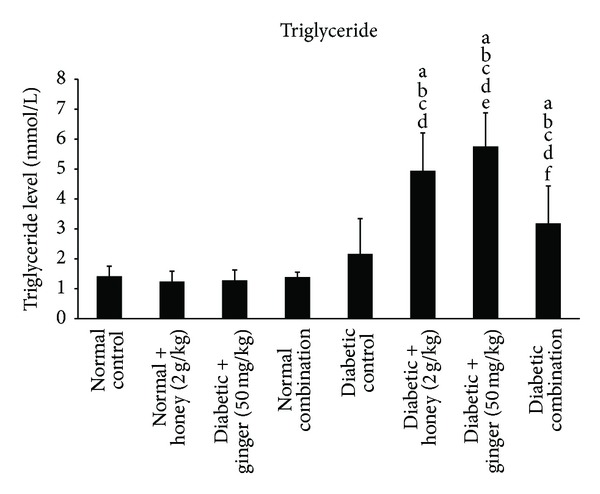
Blood triglyceride level after 3 weeks of treatment in normal and STZ-induced diabetic rats with various treatments. Data are expressed as mean ± SD. a: significant (*P* < 0.05) compared to normal control; b: significant (*P* < 0.05) compared to normal + gelam honey; c: significant (*P* < 0.05) compared to normal + ginger; d: significant (*P* < 0.05) compared to normal combination; e: significant (*P* < 0.05) compared to diabetic control; f: significant (*P* < 0.05) compared to diabetic + gelam honey.

The effect of gelam honey, ginger, and their combination on triglyceride (TG) level in STZ-induced diabetic rat is shown in Figure 2. There was a significantly (*P* < 0.05) higher level of TG in the diabetic group treated with single treatment of honey and ginger; however combination of ginger and honey significantly (*P* < 0.05) reduced the TG level compared to single treatment in diabetic rats.

### 3.4. Effect of Gelam Honey, Ginger, and Their Combination on Superoxide Dismutase (SOD) Level in STZ-Induced Diabetic Rats

**Figure 3:**
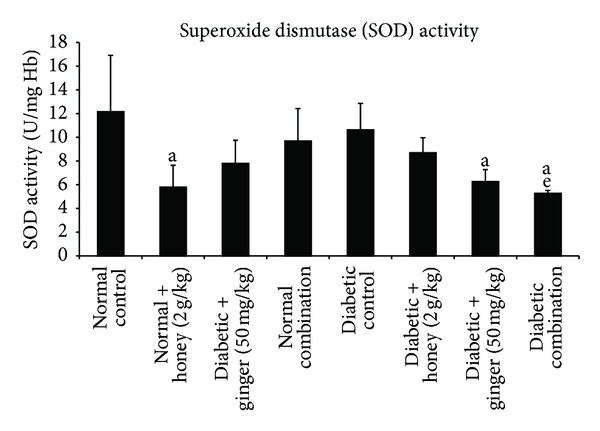
Blood superoxide dismutase (SOD) level after 3 weeks of treatment in normal and STZ-induced diabetic rats with various treatments. Data are expressed as mean ± SD. a: significant (*P* < 0.05) compared to normal control; e: significant (*P* < 0.05) compared to diabetic control.

No significant change in the SOD activity was observed between normal control and diabetic control group. In contrast, the activity of SOD enzyme decreased significantly (*P* < 0.05) in the diabetic rats treated with ginger and combination of gelam honey and ginger as compared to normal rats and diabetic control group (Figure 3).

### 3.5. Effect of Gelam Honey, Ginger, and Their Combination on Catalase (CAT) Level in STZ-Induced Diabetic Rats

**Figure 4:**
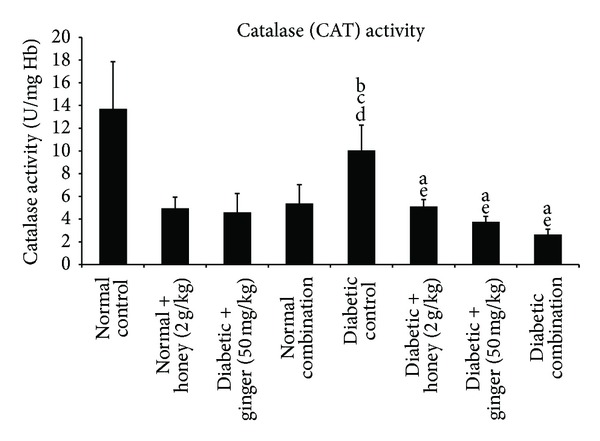
Blood catalase (CAT) level after 3 weeks of treatment in normal and STZ-induced diabetic rats with various treatments. Data are expressed as mean ± SD. a: significant (*P* < 0.05) compared to normal control; b: significant (*P* < 0.05) compared to normal + gelam honey; c: significant (*P* < 0.05) compared to normal + ginger; d: significant (*P* < 0.05) compared to normal combination; e: significant (*P* < 0.05) compared to diabetic control.

Diabetic rats had significantly (*P* < 0.05) higher levels of CAT activity compared to the nondiabetic rats with treatment. However, the diabetic group treated with gelam honey, ginger, and their combination managed to show a significant (*P* < 0.05) reduction in CAT activity when compared with the diabetic control and normal control group (Figure 4).

### 3.6. Effect of Gelam Honey, Ginger, and Their Combination on Glutathione Peroxidase (GPx) Level in STZ-Induced Diabetic Rats

**Figure 5:**
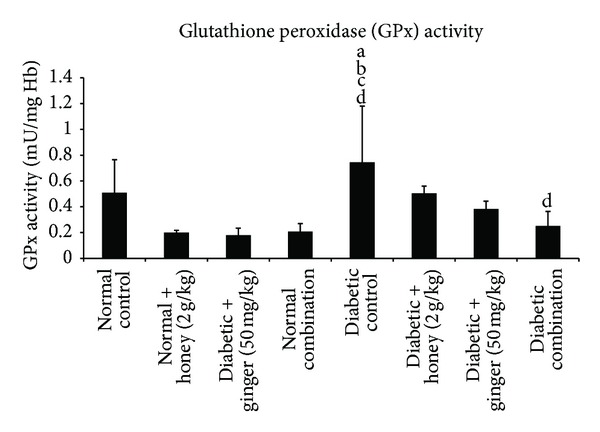
Blood glutathione peroxidase (GPx) level after 3 weeks of treatment in normal and STZ-induced diabetic rats with various treatments. Data are expressed as mean ± SD. a: significant (*P* < 0.05) compared to normal control; b: significant (*P* < 0.05) compared to normal + gelam honey; c: significant (*P* < 0.05) compared to normal + ginger; d: significant (*P* < 0.05) compared to normal combination.

Diabetic rats showed a significantly (*P* < 0.05) higher GPx activity when compared to the nondiabetic rats. Treatment of diabetic rats with gelam honey, ginger, and their combination did not produce any significant effects on GPx activity compared to normal control and diabetic control group (Figure 5).

### 3.7. Effect of Gelam Honey, Ginger, and Their Combination on Levels of Reduced Glutathione (GSH) and on GSH/GSSG Ratio in STZ-Induced Diabetic Rats

**Figure 6:**
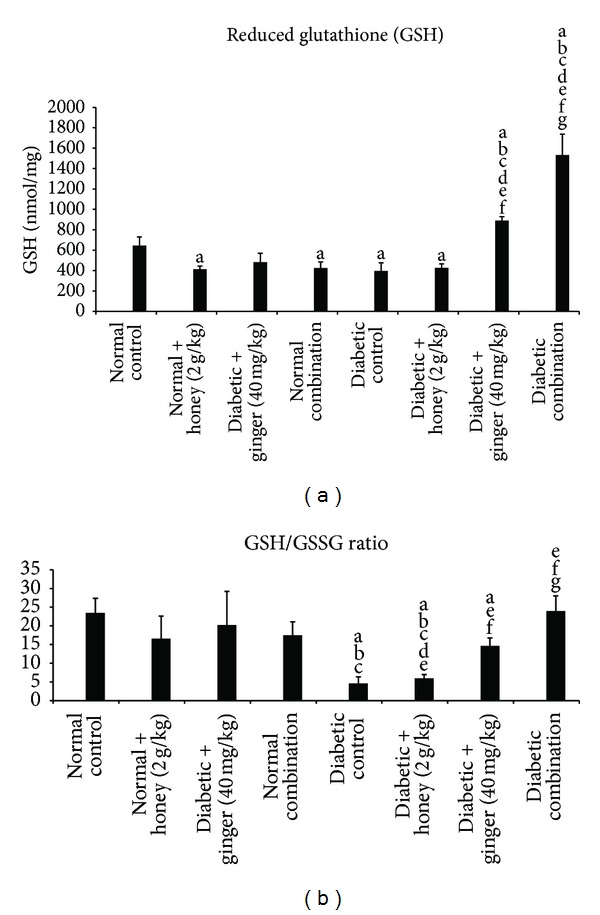
(a) Plasma GSH level and (b) ratio of GSH/GSSG after 3 weeks of treatment in normal and STZ-induced diabetic rats with various treatments. Data are expressed as mean ± SD. a: significant (*P* < 0.05) compared to normal control; b: significant (*P* < 0.05) compared to normal + gelam honey; c: significant (*P* < 0.05) compared to normal + ginger; d: significant (*P* < 0.05) compared to normal combination; e: significant (*P* < 0.05) compared to diabetic control; f: significant (*P* < 0.05) compared to diabetic + gelam honey; g: significant (*P* < 0.05) compared to diabetic + ginger.

Diabetic rats showed a significantly (*P* < 0.05) lower GSH content and GSH/GSSG ratio as compared to the nondiabetic rats. Combination treatment of gelam honey and ginger increased GSH content and GSH/GSSG ratio significantly (*P* < 0.05) when compared to the diabetic control group (Figure 6).

### 3.8. Effect of Gelam Honey, Ginger, and Their Combination on Malondialdehyde (MDA) Level in STZ-Induced Diabetic Rats

**Figure 7:**
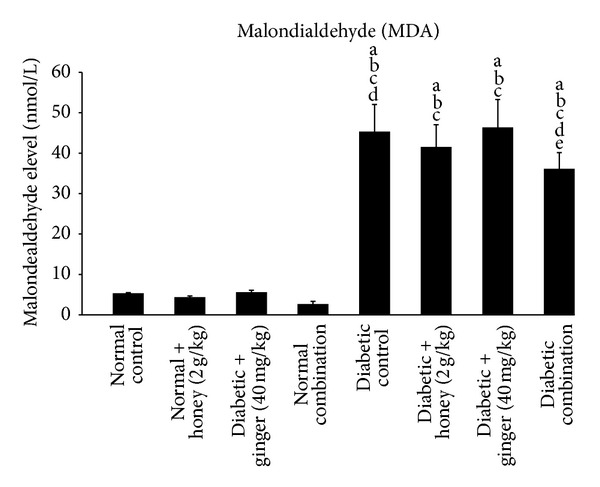
Plasma malondialdehyde (MDA) level after 3 weeks of treatment in normal and STZ-induced diabetic rats with various treatments. Data are expressed as mean ± SD. a: significant (*P* < 0.05) compared to normal control; b: significant (*P* < 0.05) compared to normal + gelam honey; c: significant (*P* < 0.05) compared to normal + ginger; d: significant (*P* < 0.05) compared to normal combination; e: significant (*P* < 0.05) compared to diabetic control.

The result demonstrated that MDA level is significantly (*P* < 0.05) increased in diabetic rats as compared to nondiabetic rats. However, the diabetic rats that received a combination of gelam honey and ginger exhibited a significant (*P* < 0.05) decrease in MDA concentration compared to diabetic control rats (Figure 7).

## 4. Discussion 

The role of oxidative stress in the pathogenesis and complications of diabetes mellitus is well recognized [28–31]. Several studies have reported that persistent hyperglycaemia can cause high production of ROS which may lead to cellular oxidative damage including DNA, lipids, and protein [32–35]. Thus, reducing the blood glucose level in DM is important to decrease the production of ROS as well as to alleviate complications of DM as a result of tissues damage. Omotayo et al. [36] showed that honey exerts a hypoglycemic effect and ameliorates the oxidative stress in kidneys of STZ induced diabetic rats after following four weeks of treatment, while Ojewole [37] found that ginger exhibits similar hypoglycaemic properties under normal and diabetic condition. The ability of ginger to reduce the blood glucose level in STZ-induced DM rats was also reported by Morakinyo et al. [38] after six weeks of treatment. However, in the present study, we did not observe any hypoglycaemic effect of honey, ginger, and their combination in diabetic rats over the three weeks of treatment. This may be due to the shorter duration of treatment adopted in this study. It was also noted that TG levels were high in diabetic control and diabetic treatment groups, showing that neither honey nor ginger had hypolipidemic effect on diabetic rats. Our results are contradictory to the study by Akhani et al. [16] and Al-Amin et al. [39] who reported that ginger extract was significantly effective in lowering glucose, cholesterol, and TG levels in STZ-induced diabetic rats compared with control diabetic rats. Perhaps, this can be explained by excessively high glucose levels that are known to convert to TG during carbohydrate metabolism [40].

Reactive oxygen species (ROS) such as hydroxyl radical (OH^∙^), superoxide anion (O_2_^∙^), and hydrogen peroxide (H_2_O_2_) are naturally generated in the body during normal metabolism. They are neutralized by the endogenous antioxidant enzymes, such as SOD, CAT, and GPx, to prevent oxidative stress [41]. However, overproduction of ROS in DM leads to imbalance between oxidants and antioxidants resulting in the development of complications of DM such as diabetic neuropathy and nephropathy [8]. Exogenous antioxidants such as ginger, honey,* Chlorella vulgaris*, vitamin E, and vitamin C may help in eradicating the large amount of ROS in DM [13, 21, 37, 42].

The antioxidant defence system enzymes SOD, CAT, and GPx are increased in DM because of excessive levels of glucose in mitochondria resulting in overdrive of electron transport chain producing excess free radicals [43]. Several studies have shown the ability of honey and ginger to modulate antioxidant enzymes activities in DM. Omotayo et al. [36] showed that SOD activity was decreased in the STZ-induced diabetic rats treated with honey. In the present study, both honey and ginger were able to reduce the SOD and CAT activities significantly in STZ-induced diabetic rats when compared to diabetic rats without treatment. Interestingly, we found that the diabetic rats treated with combination of honey and ginger showed lower SOD and CAT activities when compared to diabetic group treated with honey and ginger alone. The ability of honey to prevent oxidative damage in diabetic rats might be due to its phenolic antioxidants content or through the action of CAT activity found in honey in reducing hydrogen peroxide. Ginger extract possesses antioxidative characteristic by scavenging superoxide anion and hydroxyl radicals [44]. We also observed a nonsignificant reduction in SOD activity in diabetic group compared to normal control group.

Glutathione is the major endogenous antioxidant produced by the cells, participating directly in the neutralization of free radicals and reactive oxygen compounds [45]. In healthy cells and tissues, most of the total glutathione are in the reduced form (GSH) and less than 10% exists in the oxidized form (GSSG). The ratio of GSH to GSSG is a sensitive indicator of oxidative stress or overall health [46]. The results of the present study showed that GSH level and GSH/GSSG ratio were decreased in diabetic rats. This significant depletion of GSH level and GSH/GSSG ratio in diabetic rats would indicate its increased utilization against reactive oxygen species generated in diabetic rats [47]. Although treatment with gelam honey alone did not produce any effect on GSH level or GSH/GSSG ratio, ginger extract however managed to increase GSH level and GSH/GSSG ratio back to normal level, while the combination treatment showed significant increase above normal level. Thus, our findings suggest that combination of honey and ginger may offer better antioxidant effect by scavenging free radicals and restoring the imbalance between oxidant/antioxidant homeostasis developed during diabetic condition.

Malondialdehyde (MDA) is generated from the degradation of polyunsaturated lipids by ROS. It is one of the most frequently used indicators of lipid peroxidation. Omotayo et al. [36] had demonstrated that elevated levels of MDA in STZ-induced diabetic rats were reduced after the treatment with honey. Similarly, our study showed that the diabetic rats which received gelam honey in combination with ginger showed significant reduction in lipid peroxidation (MDA). However gelam honey and ginger alone may not offer protection against lipid peroxidative damage in diabetic rats.

## 5. Conclusion 

DM is a common chronic disease which can cause multiple complications if the glucose level is uncontrolled. This is due to the oxidative stress which can cause changes in the endogenous antioxidants. Our present study showed that the combination of gelam honey and ginger provided a better antioxidant effect as compared to gelam honey or ginger alone as evidenced by the significantly reduced SOD and CAT activities, depleted MDA level, increased GSH level, and increased GSH/GSSG ratio in diabetic rats. Further research with longer duration of treatment with ginger and honey would perhaps give better insight to the changes in the biochemical profile related to diabetic complications. The correct combination of gelam honey and ginger may offer a potential adjuvant to antidiabetic medications to reduce the oxidative stress and complications of DM.

## Figures and Tables

**Table 1:** Effects of honey, ginger and the combination of honey and ginger on body weight (g) in STZ-induced diabetic rats.

Groups	Body weight in gms (mean ± SD)			
	0 week	1st week	2nd week	3rd week
Normal Control	227.6 ± 10.7	270.2 ± 23.0^a^	295.7 ± 26.7^a,b^	301.0 ± 30.7^a,b^
Normal + Honey	231.1 ± 13.8	272.0 ± 14.1^a^	291.1 ± 16.1^a,b^	306.3 ± 15.8^a,b^
Normal + Ginger	211.2 ± 20.3	255.1 ± 31.1	271.6 ± 37.1^b^	290.8 ± 37.4^a,b,c^
Normal + Combination	226.1 ± 17.4	269.4 ± 11.5	287.6 ± 13.1^a,b^	304.2 ± 17.1^b,c^
Diabetic Control	224.8 ± 8.5	209.9 ± 17.3^a^	214.0 ± 29.6^a,b^	205.9 ± 52.5
Diabetic + Honey	230.9 ± 4.2	217.6 ± 16.2^a^	214.2 ± 22.0^a^	213.9 ± 32.3^a^
Diabetic + Ginger	233.7 ± 8.9	203.0 ± 31.6^a^	195.7 ± 30.3^a,b^	188.0 ± 31.9^a,b,c^
Diabetic + Combination	238.3 ± 11.3	213.7 ± 18.1	204.2 ± 27.3	202.2 ± 38.0^a^

Data are expressed as mean ± SD. ^a^Significant different (*P* < 0.05) when compared to week 0, ^b^significant different (*P* < 0.05) when compared to week 1, ^c^significant different (*P* < 0.05) when compared to week 2.
